# Assessment of CHADS_2_ and CHA_2_DS_2_-VASc scores in obstructive sleep apnea patients with atrial fibrillation

**DOI:** 10.1007/s11325-014-1042-5

**Published:** 2014-08-02

**Authors:** Filip M. Szymanski, Krzysztof J. Filipiak, Anna E. Platek, Anna Hrynkiewicz-Szymanska, Grzegorz Karpinski, Grzegorz Opolski

**Affiliations:** 1 Department of Cardiology, Medical University of Warsaw, 1A Banacha Street, Warsaw, 02-097 Poland; 2 Department of Cardiology, Hypertension and Internal Diseases, Medical University of Warsaw, Warsaw, Poland

**Keywords:** Atrial fibrillation, Obstructive sleep apnea, Stroke risk, CHADS_2_, CHA_2_DS_2_-VASc

## Abstract

**Purpose:**

Assessment of stroke risk and implementation of appropriate antithrombotic therapy is an important issue in atrial fibrillation patients. Current risk scores do not take into consideration the comorbidities associated with elevated thromboembolic like obstructive sleep apnea (OSA). The aim of the study was to establish whether atrial fibrillation patients with coexisting OSA have higher stroke risk according to CHADS_2_ and CHA_2_DS_2_-VASc scores.

**Methods:**

Two hundred fifty-four consecutive patients hospitalized with a primary diagnosis of atrial fibrillation participated in the study. All patients underwent whole night polygraphy and were scored in both CHADS_2_ and CHA_2_DS_2_-VASc according to their medical records or de novo diagnosis.

**Results:**

The study population was predominantly male (65.4 %; mean age, 57.5 ± 10.0 years) with a high prevalence of hypertension (73.6 %), dyslipidemia (63.4 %), and obesity (42.9 %). OSA was present in 47.6 % of patients, who more often had history of stroke (*p* = 0.0007). Stroke risk profile assessed by both CHADS_2_ and CHA_2_DS_2_-VASc scores was higher in patients with OSA (1.2 ± 0.9 vs. 0.8 ± 0.6; *p* < 0.0001 and 2.2 ± 1.7 vs. 1.5 ± 1.1; *p* = 0.001) than without it. Differences in the stroke risk remained significant across different age strata, and the trend for point values in CHADS_2_ and CHA_2_DS_2_-VASc scores rose along with OSA severity according to the apnea–hypopnea index (AHI; *p* for trend <0.001).

**Conclusions:**

OSA was highly prevalent in atrial fibrillation patients. Patients with OSA have higher CHADS_2_ and CHA_2_DS_2_-VASc scores. Mean CHADS_2_ and CHA_2_DS_2_-VASc scores rise with OSA severity. Future studies should prospectively research on potential inclusion of OSA to stroke prediction models.

## Introduction

Atrial fibrillation is one of the most common types of arrhythmia. Currently, it occurs in approximately 2 % of the general population, but it is estimated that its incidence will increase even more [1, 2]. The presence of the arrhythmia is associated with an elevated risk of cardiovascular events, most of all stroke [3]. It is estimated that approximately one fifth of all strokes is attributable to the atrial fibrillation and, therefore, could be avoided if patients were managed properly [4]. Given the high atrial fibrillation prevalence and disabling consequences of stroke, prevention of the thromboembolic events is currently one of the key issues in treatment of atrial fibrillation patients [3, 5].

Current guidelines recommend planning the anticoagulation treatment individually, according to the patients’ thromboembolic risk. Two tools recommended to be used in this indication are the CHADS_2_ and CHA_2_DS_2_-VASc scores. Both tools are based on easy to obtain clinical data including the presence of congestive heart failure, age, diabetes mellitus, sex, history of stroke, or vascular disease. Point values obtained in the CHADS_2_ and CHA_2_DS_2_-VASc scores give us approximate information of the annual stroke risk and, therefore, indications for anticoagulation treatment [6, 7].

Unfortunately, like most point scores, CHADS_2_ and CHA_2_DS_2_-VASc are likely to overlook important parameters, including comorbidities associated with elevated thromboembolic risk. One of them is obstructive sleep apnea (OSA), a sleep-disordered breathing with high prevalence in atrial fibrillation patients (estimated to be between 32 and 49 %) [8]. OSA is strongly associated with hypercoagulation, which has been reported in the literature previously [9–11]. OSA influence strongly also on prevalence of hypertension, sympathetic dysregulation, or many other cardiovascular risk factors, which causes elevated risk of stroke and other thromboembolic events. The strongly elevated thromboembolic risk and mutual exacerbation of the diseases even caused that, recently, atrial fibrillation and OSA have been postulated to be components of one clinical syndrome [12].

We hypothesize that the stroke risk will be higher in patients with OSA and that this relationship will be clearly shown by the results of CHADS_2_ and CHA_2_DS_2_-VASc scores in both groups of patients. The aim of the study was to determine if the occurrence of OSA in atrial fibrillation patients is associated with higher stroke risk assessed by CHADS_2_ and CHA_2_DS_2_-VASc scores.

## Methods

### Study population

In accordance with the Declaration of Helsinki, the study protocol was approved by the Regional Ethics Committee. Before the study entry, a written informed consent was obtained from every study participant. Two hundred fifty-four consecutive patients hospitalized in a high-volume, tertiary University Hospital Cardiology Department were enrolled. The cause of admission in every patient was prequalification for atrial fibrillation ablation procedure. Subjects were eligible to participate in this study if they were 18 to 75 years of age without previously diagnosed OSA or central sleep apnea and no current treatment with continuous positive airway pressure (CPAP) device. Exclusion criteria included prior ablation for atrial fibrillation, untreated overt hyper- or hypothyroidism (defined according to current guidelines), myocardial infarction or decompensation of heart failure within 6 months prior to study entry, fatal condition with estimated life expectancy of ≤6 months, presence of contraindications for polygraphy study including acute and/or chronic pulmonary diseases like obstructive pulmonary disease or active tuberculosis, neuromuscular disease, hemochromatosis, severe neurologic, or psychiatric disorders [13, 14].

### Assessment of atrial fibrillation and cardiovascular risk

Medical history of patients, including occurrence of cardiovascular risk factors, was taken on admission in every patient by a qualified physician. In the night following the admission, every patient underwent a whole night sleep study using a portable polygraphy device for diagnosing sleep disorder breathing (Embletta Gold; Flaga, Reykjavik, Iceland).

Diagnosis of atrial fibrillation was based on at least one arrhythmia episode recorded in a 24-h ECG Holter monitoring in 6 months prior to the study enrollment. Diagnosis of the arrhythmia was determined with respect to the European Society of Cardiology Guidelines for the management of atrial fibrillation from 2010 and its update from 2012 [3, 5]. Patients included in the study were prequalified for atrial fibrillation ablation; therefore, no permanent atrial fibrillation was diagnosed in the study population (permanent arrhythmia is diagnosed only if it is decided not to pursue rhythm control strategy). Paroxysmal atrial fibrillation was defined as self-terminating, usually within 48 h, which may continue for up to 7 days. Persistent atrial fibrillation was defined when arrhythmia episode either lasted longer than 7 days or required termination by cardioversion either with drugs or by direct current cardioversion [3].

All patients were interviewed for history of cardiac failure, moderate to severe left ventricle systolic dysfunction, hypertension, diabetes, stroke or transient ischemic attack (TIA), and vascular disease (including myocardial infarction, complex aortic plaque, and peripheral artery disease including prior revascularization, amputation due to peripheral artery disease, or its angiographic evidence). Diagnosis of the abovementioned diseases were made basing on eligible medical records, taking prescription drugs applicable for the respective disease (i.e., hypoglycemic agents for diabetes) or as a de novo diagnosis according to current criteria.

### Assessment of stroke risk

The CHADS_2_ and CHA_2_DS_2_-VASc scores were calculated for every patient based on predefined point systems according to the current guidelines for scoring and diagnosing all mentioned conditions [6, 7]. In the CHA_2_DS_2_-VASc score, 2 points were assigned if a patient had a history of stroke or TIA or was ≥75 years of age. One point was assigned for the age 65–74 years, female sex, history of hypertension, diabetes, recent cardiac failure, and vascular disease (history of myocardial infarction, presence complex aortic plaque, or peripheral artery disease). In the CHADS_2_ score, 2 points are assigned for the history of stroke or TIA, and 1 point for each of the following: history of congestive heart failure, diabetes or hypertension, and age ≥75 years.

### Diagnosis of OSA

Polygraphy used in the study is a level 3 sleep monitoring tool according to the recommendations of the European Respiratory Society and the European Society of Hypertension [15]. This kind of devices record >4 channels including channels to detect respiratory movements or respiratory effort, airflow, heart rate, ECG, and oxygen saturation and are used for objective OSA diagnosis confirmation. All polygraphy results were scored manually according to the recommendations of the American Academy of Sleep Medicine by a qualified cardiologist specialized in sleep medicine [16]. Apnea was defined as a complete cessation (residual air flow equal of <10 % of the preceding stable breathing period) of airflow lasting ≥10 s. Obstructive breathing event occurs with complete upper airway obstruction. Hypopnea was defined as a noticeable decrease (>30 and <90 %) in the respiratory signal amplitude which lasted for >10 and accompanied by desaturation (≥4 %). OSA diagnosis was made when the apnea–hypopnea index (AHI, number of apneas and hypopneas per hour) was ≥5 per hour. Patients with confirmed OSA were subsequently grouped according to OSA severity with AHI ≥ OSA severity was classified as mild when AHI ≥5 and <15 per hour, moderate when AHI was ≥15 and ≤30 per hour, and as severe when AHI was >30 per hour.

### Statistical analysis

Data were tested for normality using the Kolmogorov–Smirnov test. Continuous data are presented as mean and 95 % confidence intervals (CI), with statistical comparisons performed with the Mann–Whitney test or Student’s *t* test. Categorical variable comparison was made using either the chi-square or Fisher exact tests. A one-way analysis of variance was used to assess the impact of OSA severity according to AHI on the CHADS_2_ and CHA_2_DS_2_-VASc scores. A *p* value of less than 0.05 was considered statistically significant, whereas the confidence intervals were 95 %. All statistical calculations were performed using commercially available SAS statistical software version 8.02 (SAS Institute, Inc., Cary, NC, USA).

## Results

The baseline characteristics of the study population are described in Table 1. Of the 254 patients, 88 (34.6 %) were females. Mean age of the study population was 57.5 ± 10.0 years. Hypertension was present in 73.6 % of patients, whereas 63.4 % suffered from dyslipidemia. The prevalence of body mass index (BMI) exceeding criteria for obesity diagnosis (>30 kg/m^2^) was 42.9 %. Paroxysmal atrial fibrillation was present in the majority of patients (69.3 %), and for the rest, persistent atrial fibrillation was diagnosed. Mean CHADS_2_ and CHA_2_DS_2_-VASc scores were, respectively, 1.0 ± 0.8 and 1.8 ± 1.4. Mean AHI value in the whole study population was 8.4 ± 10.8 per hour.

**Table 1:** Baseline characteristics of the study population

Parameter	Value
Age (years)	57.5 ± 10.0
Female	88 (34.6 %)
Hypertension	187 (73.6 %)
Diabetes mellitus	23 (9.1 %)
Prior stroke or TIA	18 (7.1 %)
Dyslipidemia	161 (63.4 %)
Smoking	26 (10.2 %)
SBP (mm Hg)	132.1 ± 16.9
DBP (mm Hg)	80.9 ± 11.1
BMI >30 kg/m^2^	109 (42.9 %)
Neck circumference (cm)	40.3 ± 3.6
Waist circumference (cm)	108.4 ± 65.7
Paroxysmal AF	176 (69.3 %)
AHI <5 per hour	133 (52.4 %)
AHI ≥5 and <15 per hour	74 (29.1 %)
AHI ≥15 and <30 per hour	35 (13.8 %)
AHI ≥30 per hour	12 (4.7 %)
CHADS_2_	1.0 ± 0.8
CHA_2_DS_2_-VASc	1.8 ± 1.4
AHI (per hour)	8.4 ± 10.8

Values are mean ± SD or *n* (%),

*AF* atrial fibrillation, *AHI* apnea–hypopnea index, *BMI* body mass index, *DBP* diastolic blood pressure, *SBP* systolic blood pressure, *SD* standard deviation, *TIA* transient ischemic attack

OSA was present in 47.6 % of patients. Details of patients’ characteristics according to OSA status are presented in Table 2. Patients with OSA were older (59.6 ± 7.9 vs. 55.5 ± 11.3 years; *p* = 0.008) and had higher neck (41.2 ± 3.8 vs. 39.4 ± 3.3 cm; *p* = 0.0002) and waist circumferences (108.5 ± 13.1 vs. 108.3 ± 89.2 cm; *p* < 0.0001) than patients without OSA. Obesity was diagnosed in more patients in OSA than non-OSA group (54.5 vs. 32.3 %; *p* = 0.0006). Also, the prevalence of other comorbidities was different in patients with and without OSA. Patients with OSA more often were diagnosed with hypertension (80.2 vs. 67.7 %; *p* = 0.03), diabetes mellitus (13.2 vs. 5.3 %; *p* = 0.05), or had history of stroke (13.2 vs. 1.5 %; *p* = 0.0007) than patients without the sleep-disordered breathing condition. The form of arrhythmia that was predominant in OSA patients was persistent atrial fibrillation, whereas in patients without OSA, atrial fibrillation was more often paroxysmal (*p* = 0.02).

**Table 2:** Patients’ characteristics according to the presence of obstructive sleep apnea

Parameter	Patients without OSA (*n* = 133)	Patients with OSA (*n* = 121)	*p* value
Age (years)	55.5 ± 11.3	59.6 ± 7.9	0.008
Female	52 (39.1 %)	36 (29.8 %)	0.15
Hypertension	90 (67.7 %)	97 (80.2 %)	0.03
Diabetes mellitus	7 (5.3 %)	16 (13.2 %)	0.05
Prior stroke or TIA	2 (1.5 %)	16 (13.2 %)	0.0007
Dyslipidemia	84 (63.2 %)	77 (63.6 %)	0.96
Smoking	16 (12.0 %)	10 (8.3 %)	0.43
SBP (mm Hg)	131.5 ± 17.0	132.8 ± 16.8	0.55
DBP (mm Hg)	80.0 ± 10.8	82.0 ± 11.5	0.16
BMI >30 kg/m^2^	43 (32.3 %)	66 (54.5 %)	0.0006
Neck circumference (cm)	39.4 ± 3.3	41.2 ± 3.8	0.0002
Waist circumference (cm)	108.3 ± 89.2	108.5 ± 13.1	<0.0001
Paroxysmal AF	101 (75.9 %)	75 (62.0 %)	0.02
CHADS_2_	0.8 ± 0.6	1.2 ± 0.9	<0.0001
CHA_2_DS_2_-VASc	1.5 ± 1.1	2.2 ± 1.7	0.001

Values are mean ± SD or *n* (%)

*AF* atrial fibrillation, *AHI* apnea–hypopnea index, *BMI* body mass index, *DBP* diastolic blood pressure, *OSA* obstructive sleep apnea, *SBP* systolic blood pressure, *SD* standard deviation, *TIA* transient ischemic attack

**Fig. 1:**
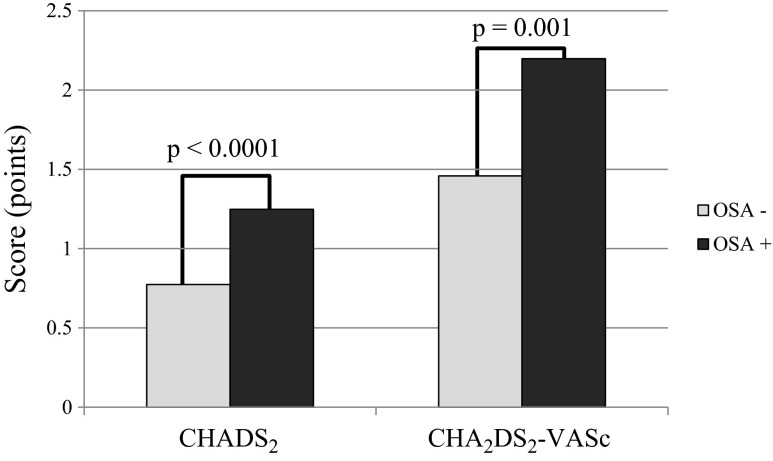
Mean CHADS_2_ and CHA_2_DS_2_-VASc scores in patients with and without obstructive sleep apnea (OSA)

**Fig. 2:**
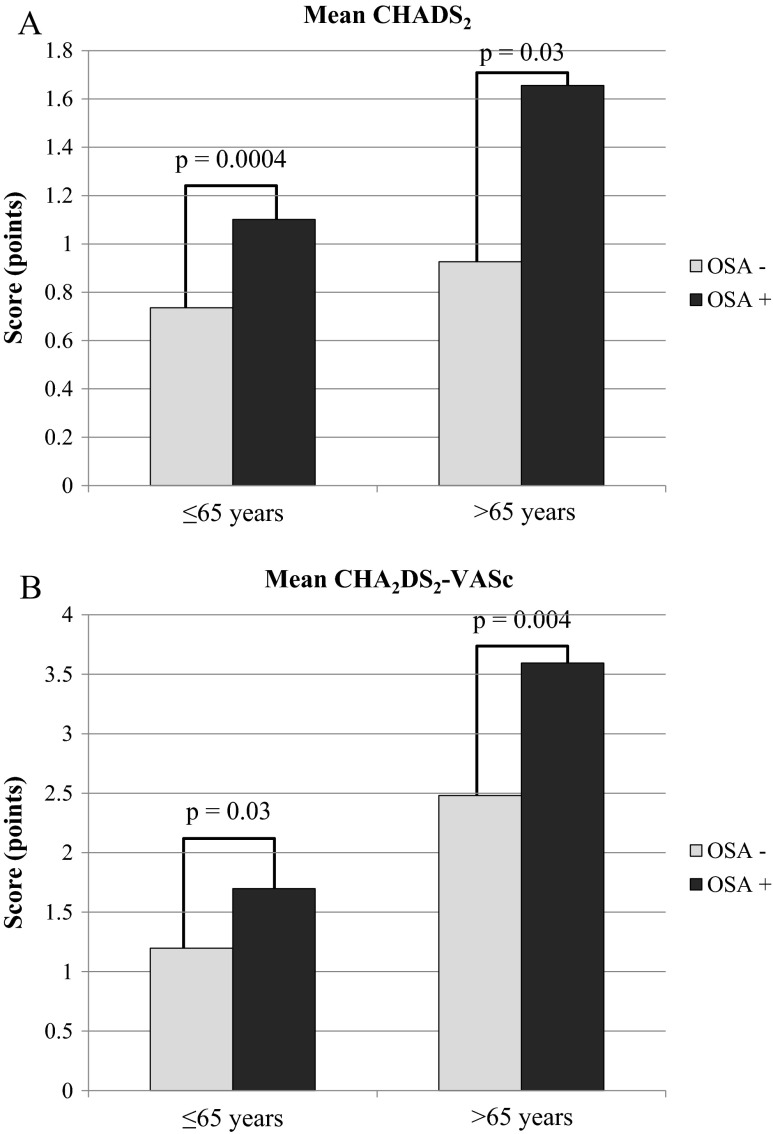
Relationship of mean obstructive sleep apnea (OSA) with CHADS_2_ (**a**) and CHA_2_DS_2_-VASc (**b**) scores in different age strata

General thromboembolic risk profile assessed by both CHADS_2_ and CHA_2_DS_2_-VASc scores was higher in patients with OSA than without it. Mean CHADS_2_ score in the group with and without OSA was 1.2 ± 0.9 and 0.8 ± 0.6 points, respectively (*p* < 0.0001). In CHA_2_DS_2_-VASc scale, patients with OSA scored 2.2 ± 1.7, while the mean score in non-OSA patients was 1.5 ± 1.1 points (*p* = 0.001) (Fig. 1). Differences in thromboembolic risk remained significant even when we analyzed patients in different age strata. CHADS_2_ score in patients ≤65 years old was higher in OSA than non-OSA patients (0.7 ± 0.6 vs. 1.1 ± 0.8 points; *p* = 0.0004), similarly to patients >65 years of age (0.9 ± 0.5 vs. 1.7 ± 1.3 points; *p* = 0.03, respectively) (Fig. 2a). The same trend was observed in respect to the mean CHA_2_DS_2_-VASc score. In patients ≤65 years old, the mean value in OSA patients was 1.2 ± 0.9 and in non-OSA patients was 1.7 ± 1.4 points (*p* = 0.03). Analysis of the group of patients older than 65 years also showed that OSA patients had higher (2.5 ± 1.0 points) CHA_2_DS_2_-VASc score than non-OSA patients (3.6 ± 1.54 points; *p* = 0.004) (Fig. 2b).

**Fig. 3:**
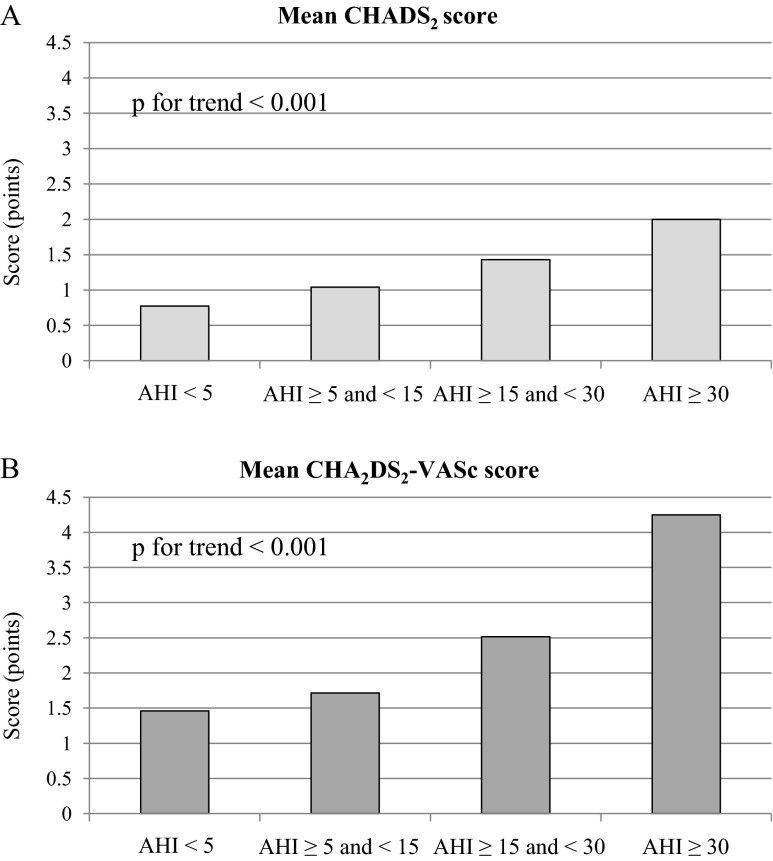
Trends in CHADS_2_ (**a**) and CHA_2_DS_2_-VASc (**b**) scores according to obstructive sleep apnea (OSA) severity

In the general population, both CHADS_2_ and CHA_2_DS_2_-VASc scores seemed to correspond with OSA severity. Dividing the patients into four groups: non-OSA (with AHI <5 per hour), mild OSA (AHI ≥5 and <15 per hour), moderate OSA (AHI ≥15 and ≤30 per hour), and severe OSA (AHI >30 per hour) showed that there was a significant trend towards a rise in CHADS_2_ (*p* for trend <0.001) and CHA_2_DS_2_-VASc scores (*p* for trend <0.001) (Fig. 3).

To perform a sensitivity analysis, we divided patients into groups with paroxysmal and permanent atrial fibrillation. We observed higher CHADS_2_ and CHA_2_DS_2_-VASc scores in all OSA patients. In permanent atrial fibrillation patients, the mean CHADS_2_ score was 0.9 ± 0.5 vs. 1.2 ± 0.8 (*p* = 0.3) for non-OSA and OSA patients, respectively, and CHA_2_DS_2_-VASc score was 1.7 ± 1.0 vs. 1.9 ± 1.6 (*p* = 0.8). In the group with paroxysmal atrial fibrillation, the mean CHADS_2_ was 0.7 ± 0.6 vs. 1.3 ± 1.0 (*p* < 0.0001), and the mean CHA_2_DS_2_-VASc was 1.4 ± 1.1 vs. 2.4 ± 1.7 (*p* < 0.0001) respectively.

## Discussion

CHADS_2_ and CHA_2_DS_2_-VASc scores are simple risk assessment schemes. CHADS_2_ was developed basing on the AF Investigators and Stroke Prevention in Atrial Fibrillation (SPAF) Investigators criteria and includes stroke risk factors like recent cardiac failure, hypertension, age >75 years, diabetes, and history of stroke or TIA [6]. The presence of those factors showed to be predictive for thromboembolic risk in the population of SPAF trial [17]. In all of the SPAF trials, OSA was not assessed and, therefore, could not be included in the risk model. Risk assessment scheme was developed with data from Euro Heart Survey on Atrial Fibrillation, which again does not include data on OSA [18]. CHA_2_DS_2_-VASc score attributes points for history of congestive heart failure, hypertension, diabetes, stroke, vascular disease, age ≥75 or 65–74, and female sex [7]. Both scores showed to predict incidence of stroke basing on the patient’s score [6, 19]. Moreover, they showed value in predicting other complications like pulmonary embolism or coronary artery disease severity [20, 21]. Some authors even postulated that CHADS_2_ can be useful in predicting ischemic stroke in patients without atrial fibrillation [22]. Both scores are clinically useful, but there are several disparities between them. Current guidelines encourage the use of CHA_2_DS_2_-VASc because some analyses showed that CHADS_2_ may underestimate stroke risk in patients with lower value range [23]. Nevertheless, both schemes are not free from flaws, and both may underestimate real stroke risk.

Meta-analyses of prospective cohort studies show that OSA patients, similarly to atrial fibrillation patients, are more likely to suffer from stroke [9–11, 24]. Moreover, sleep-disordered breathing is a predictor of all-cause mortality and recurrent vascular events following stroke [25]. In our study, like in the previous observations, the occurrence of OSA was associated with older age and obesity. These two risk factors are associated not only with the occurrence of OSA but also the promotion of atrial fibrillation in the general population [2, 26, 27]. There is a blurred line between concepts that OSA promotes atrial fibrillation, atrial fibrillation contributes to OSA development, or both conditions are caused by other common risk factors [8]. Nevertheless, cardiovascular risk, including stroke risk, is altered additively by both diseases.

The concept of adding OSA as a stroke risk factor to the CHADS_2_ has been previously proposed by Yazdan-Ashoori and Baranchuk [28], but literature lacked evidence from trials that were designed to support the thesis. It was proposed to include OSA in the CHADS_2_ score by allocating 1 point for OSA, and as the authors said, to rename the score CHADSS_2_ by adding an extra “S” for sleep apnea. The design of the present trial also does not provide an answer if OSA is an independent risk factor for future stroke and/or mortality in this group of patients, but it highlights two problems. First of all, in our study, OSA patients had higher CHADS_2_ and CHA_2_DS_2_-VASc scores even irrespectively of age group. This is caused by higher morbidity rates and worse general risk profile in this group of patients. Second of all, OSA is a strong stroke risk factor. Patients afflicted with OSA already are at higher risk of stroke when we do not incorporate OSA as an additional factor in the risk prediction model; it is possible that we can oversee some OSA patients with low CHADS_2_ and CHA_2_DS_2_-VASc scores who would benefit from anticoagulation therapy.

The newest data show that in spite of the current guidelines, many patients with atrial fibrillation in whom anticoagulation is required do not receive proper treatment in many European countries [29, 30]. In many cases, patients with atrial fibrillation are undertreated, and many of the strokes associated with OSA could have been avoided by proper risk assessment and more aggressive treatment. Some authors even propose for active screening for atrial fibrillation in all OSA patients, saying that in this high-risk group, this kind of intervention would benefit in stroke rate reduction [31]. OSA screening is easy and widely applicable, while confirmation of the diagnosis with polygraphy or polysomnography becomes more accessible.

In conclusion, OSA was highly prevalent in atrial fibrillation patients. Patients with OSA have higher CHADS_2_ and CHA_2_DS_2_-VASc scores. Mean CHADS_2_ and CHA_2_DS_2_-VASc scores rise with OSA severity assessed by AHI. OSA can be a predictor of higher stroke risk in atrial fibrillation patients. All atrial fibrillation patients with OSA should be monitored more carefully for stroke risk factors and promptly introduced with anticoagulation therapy if needed. Further studies are needed to decide if OSA carries a risk beyond its most often comorbidities incorporated in the CHADS_2_ and CHA_2_DS_2_-VASc scores and if it should be included in the risk prediction schemes.
